# A survey of health professions students for knowledge, attitudes, and confidence about tuberculosis, 2005

**DOI:** 10.1186/1471-2458-7-219

**Published:** 2007-08-28

**Authors:** Marguerite Jackson, Shawn Harrity, Helene Hoffman, Antonino Catanzaro

**Affiliations:** 1 National Tuberculosis Curriculum Consortium (NTCC), University of California San Diego School of Medicine, San Diego, California 92103-8374, USA

## Abstract

**Background:**

In 2003 the NIH perceived a need to strengthen teaching about tuberculosis (TB) to health professions students. The National Tuberculosis Curriculum Consortium (NTCC) was funded to meet this need. The purpose of this study was to survey students enrolled in NTCC schools prior to NTCC-developed educational materials being made available to faculty.

**Methods:**

A self-administered survey for students in NTCC schools to establish a baseline level of knowledge, attitudes, and confidence about tuberculosis.

**Results:**

1480/2965 (50%) students in 28 programs in 20 NTCC schools completed the survey. If public health students are eliminated from totals (only 61 respondents of 765 public health students), the overall response proportion for the seven clinically-related disciplines was 64.5%. The majority (74%) were in schools of medicine (MD/DO), undergraduate nursing (BSN), and pharmacy (PharmD); others were in programs for physician assistants (PA), advanced practice nursing (NP/APN), respiratory therapy (RT), clinical laboratory sciences (MT/CLS), and public health (MPH). Almost 90% had attended at least one lecture about TB. Although 91.4% knew TB was transmitted via aerosols, about one-third did not know the method for administering tuberculin, or that Bacillus Calmette-Guerin (BCG) vaccine was not a contraindication to TB skin testing. Fewer than two-thirds knew that about 10% of people in the U.S.A. who have latent tuberculosis infection (LTBI) and a normal immune system will develop TB disease, or that BCG is not part of the routine vaccination program in the U.S.A. because it complicates surveillance for new TB infection.

**Conclusion:**

There is room for improvement in knowledge, attitudes, and confidence about TB by health professions students surveyed. The NTCC-developed educational products may be used by faculty to improve student performance to be assessed with future surveys.

## Background

Between 1985 and 1992, there was a dramatic increase in the incidence of tuberculosis (TB) in the United States, and the epidemiology of TB changed [1]. In retrospect, it became clear that this increase was due, in part, to transmission of TB in hospitals and prisons, and that health care workers, in addition to physicians, were at risk for infection [2-4]. Although the number of cases and incidence of TB decreased substantially over the next decade and are now decreasing at a much slower rate (e.g., 14,515 cases in 2004 compared to 14,097 cases in 2005), the proportion of TB cases in foreign-born people continues to increase (29% in 1993; 55% in 2005) and is a focus for surveillance and control efforts in the U.S. [5]. In recent years, treatment and prevention of TB has shifted from inpatient to outpatient settings. Much of the care is provided by TB specialists in public health departments and by physicians in general practices who deal with TB in the context of delivering broader services. There has also been a growing trend for health care to be provided by non-physician-clinicians [6]. There is increasing evidence that non-physician clinicians in many disciplines can provide high-quality care, with the strongest evidence derived from care at the least complex end of the clinical spectrum [7,8]. Team care models with respiratory therapists and pharmacists are increasingly more common in critical care settings [9]. Despite the shared functions, these professionals receive very different training, both in the U.S.A. and internationally.

Since the resurgence of TB almost two decades ago, there has been increasing concern about education and competence of healthcare providers to deal with specialized issues of TB prevention and treatment. Many educational materials on all aspects of TB are produced by national and international agencies such as the Centers for Disease Control and Prevention, the National Institute of Allergy and Infectious Diseases, the World Health Organization, and the International Union Against Tuberculosis and Lung Diseases; health departments; educational and research institutions; and non-profit organizations such as the American Thoracic Society. With the wealth of materials on all content areas of TB, it is disconcerting that clinicians still make frequent errors in TB treatment [10] and there is an expressed need to educate prospective and practicing healthcare workers on TB infection management and control [11].

A major step in improving TB education in the U.S. involved the creation of the National Tuberculosis Curriculum Consortium (NTCC) consisting of over 40 multi-disciplinary educators and leaders in 25 academic institutions and affiliates throughout the U.S. (***Appendix***). The NTCC is funded by the National Heart, Lung, and Blood Institute of the National Institutes of Health for five years (10/03 – 9/08). The goal of the NTCC is to improve knowledge of tuberculosis in students in health professions schools throughout the U.S.A. The NTCC is organized around sets of competencies for students in eight health disciplines: medicine (MD/DO) [12], undergraduate nursing (BSN) [13], advanced practice nursing (NP/APN), physician assistants (PA), pharmacy (PharmD), respiratory therapy (RT), clinical laboratory sciences (MT/CLS), and public health (MPH). Consortium members representing each discipline are charged with developing products that focus on the discipline-specific competencies. These products will be used by faculty in NTCC schools to teach about TB. The materials are also available via the NTCC website [14] to faculty in other schools as well as organizations that provide continuing education and lifelong learning.

To assess students for basic knowledge of TB and how confident they feel about taking care of persons with latent tuberculosis infection (LTBI) or active TB, a Student TB Survey was developed and administered in 2005. The purpose of the initial survey described in this article was to provide a baseline level of knowledge, attitudes, and confidence about tuberculosis by surveying students enrolled in NTCC schools prior to NTCC-developed educational materials being made available to faculty.

## Methods

### Study population

A total of 2965 students in 28 programs in 20 NTCC schools were potential subjects for the study.

### Instrument development

The survey instruments were designed by members of the NTCC using materials that included content and questions from two Centers for Disease Control and Prevention publications, *The Core Curriculum on Tuberculosis *[15] and the *Self-Study Modules on Tuberculosis *[16]. In addition, questions from a TB survey administered to medical students at the University of Southern California (USC) in 2002 were used as a starting point. These questions were developed by USC infectious diseases faculty in collaboration with the TB Control Officer for Los Angeles County and were administered to about 150 medical students by a graduate student in the Master of Public Health Degree Program at California State University, Northridge (L. Acevedo and B. Jones, personal communication). Additional demographic, background, and confidence questions were developed by NTCC members. Unique versions of the survey were developed for each of the 8 disciplines (medicine [MD/DO], undergraduate nursing [BSN], pharmacy [PharmD], physician assistants [PA], advanced practice nursing [NP/APN], respiratory therapy [RT], clinical laboratory sciences [MT/CLS], and public health [MPH]) with the first 18 questions common to all versions. Up to 35 additional questions were customized for each discipline. To ensure anonymity of participants, questions about age, gender, ethnicity, and country of birth were excluded. Throughout the process of instrument development, the questions were frequently reviewed and critiqued by NTCC members for clarity and face validity; however, formal instrument development procedures were not employed due to time requirements to complete the baseline survey prior to availability of NTCC-developed educational materials.

### Survey administration

Prior to administration, a master protocol was submitted to and approved by the University of California San Diego Institutional Review Board (IRB). The master protocol and UCSD approval letter were made available to NTCC members via the NTCC website [14] for use in seeking review and approval by their own IRBs. Individual answer sheets were prepared by the UCSD School of Medicine Office of Educational Computing (EdCom) to include unique identifiers that did not identify students by name or student number, but were necessary for computer scoring. The limited demographic information and coding scheme were approved by the UCSD IRB as consistent with waiving the requirement for individual signed consent. Packets of individualized coded answer sheets were sent to each participating faculty member who was responsible for producing copies of the cover sheet explaining the survey and the survey instrument. Surveys were administered prior to September 15, 2005 to provide a baseline before faculty began using NTCC-developed educational materials. In almost all cases, surveys were administered during classroom sessions where students were together for a course. This approach worked well for the clinical disciplines; however, in the three participating public health programs, students were not all together at the same time. Accordingly, MPH students in these schools were invited to participate in the survey on a voluntary basis by individually obtaining a copy of the survey and answer sheet; only 61 students chose to participate in this manner. The majority of respondents were within six or fewer months of completion of their studies. After surveys were administered, answer sheets were returned to UCSD EdCom for scoring. Item analyses were provided to each faculty member for students in their own school; item analyses for schools in the same discipline were aggregated and distributed to discipline-group members.

## Results

Data reported in this article are limited primarily to responses to the 18 questions common across all eight versions of the survey administered to students in 28 programs in 20 NTCC schools.

Of the 2965 students enrolled in the 28 programs surveyed, about half (1480) completed the survey. Table 1 shows student participation by discipline. The smallest proportion of participants was from public health. When those respondents are excluded, almost two-thirds (64.5%) of students in the seven clinically-related disciplines completed surveys. Almost three-quarters (74%) of respondents were in three disciplines: medicine, pharmacy, and undergraduate nursing.

**Table 1:** Student participation in NTCC Survey, 2005

**Discipline**	**Number of Programs**	**Total Students N**	**Respondents N (%)**
Medicine (MD/DO)	5	675	485 (72)
Physician Assistant (PA)	2	120	100 (83)
Nursing (BSN)	5	555	251 (45)
Advanced Practice Nursing (NP/APN)	4	220	92 (42)
Pharmacy (PharmD)	3	503	364 (72)
Respiratory Therapy (RT)	3	42	42 (100)
Clinical Laboratory Sciences (MT/CLS)	3	85	85 (100)
Public Health (MPH)	3	765	61 (8)*

Totals	28	2965	1480 (50.0)

*If public health students are eliminated from totals, overall response proportion for the seven clinically-related disciplines is 64.5%

Table 2 presents responses to selected questions. Almost 90% of respondents had attended at least one lecture where TB was a primary focus (NTCC faculty reported common topics were epidemiology, diagnosis, treatment, and other content pertinent to their specific discipline), and over half (56.3%) had attended three or more hours of lecture/instruction on TB. The major teaching modalities were lectures and case discussion at conferences or case studies; very few students had participated in other forms of active-learning (i.e., seminars, standardized patients, games, computer simulations, computer modules).

**Table 2:** Selected questions and responses for NTCC Survey (n = 1480)

**Question/Responses**	**N (%)**
***1. During your academic program to date, have you attended at least one lecture where tuberculosis (TB) was a primary focus?***	
• Yes	1307 (88.3)
***2. Approximately how many hours of lecture/instruction on TB have you attended?***	
• None	61 (4.1)
• 1–2 hours	585 (39.5)
• 3–4 hours	528 (35.7)
• 5–6 hours	165 (11.1)
• More than 6 hours	141 (9.5)
***3. From which of the following teaching modalities have you received TB education? (mark all that apply)***	
• None	36 (2.4)
• Lecture	1391 (94.0)
• Case discussion at conference or case study	683 (46.1)
• Seminar	174 (11.8)
• Standardized patient	149 (10.1)
• Game show format (e.g., Millionaire or Jeopardy)	22 (2.2)
• Computer simulation	36 (2.4)
• Completion of a module on a computer, followed by a series of questions answered on-line (computer based learning)	75 (5.1)
• Other	142 (9.6)
***4. Have you sought out and independently reviewed additional information about TB beyond requirements for a class or seminar?***	
• Yes	636 (43.0)
***5. Approximately how much total time have you spent outside of class learning about TB?***	
• None	354 (23.9)
• 1–2 hours	655 (44.3)
• 3–4 hours	286 (19.3)
• 5–6 hours	74 (5.0)
• More than 6 hours	109 (7.4)
**General Knowledge Questions about Tuberculosis**	
***6. Tuberculosis organisms are most commonly transmitted from person-to-person in which one of the following ways?***	
• A. Blood and body fluids	95 (6.4)
• B. Aerosol *(correct answer)*	1352 (91.4)
• C. Food	8 (0.5)
• D. Fomites	20 (1.4)
***7. What is the currently recommended method for administering tuberculin?***	
• A. Intradermal injection (Mantoux) *(correct answer)*	959 (64.8)
• B. Multi-prong method (Tine)	52 (3.5)
• C. Subcutaneous injection	437 (29.5)
• D. Inhalation	31 (2.1)
***8. Which of the following is a contraindication to TB skin testing?***	
• A. BCG vaccination	519 (35.1)
• B. TB disease	388 (26.2)
• C. Malnutrition	44 (3.0)
• D. None of the above *(correct answer)*	526 (35.5)
***9. Generally, what percentage of people in the U.S. who have LTBI and a normal immune system will go on to develop TB disease at some point in their lives?***	
• A. 1%	364 (24.6)
• B. 10% *(correct answer)*	944 (63.8)
• C. 50%	140 (9.5)
• D. 90%	29 (2.0)
***10. Why is BCG NOT PART of the routine vaccination program in the United States?***	
• A. The side effects are too severe	210 (14.2)
• B. BCG is only effective for preventing adult pulmonary TB	115 (7.8)
• C. BCG vaccination complicates surveillance for new TB infection (LTBI) *(correct answer)*	945 (63.9)
• D. There is limited experience with BCG use in children	188 (12.7)

Although almost all students knew TB was most commonly transmitted from person-to-person via aerosols (Question 6), about one-third did not know the correct method for administering tuberculin (Question 7) or that BCG was not a contraindication to TB skin testing (Question 8). Fewer than two-thirds knew that about 10% of people in the U.S. who have LTBI and a normal immune system will go on to develop TB at some point in their lives (Question 9), or that the reason BCG is not part of routine vaccination programs in the U.S. is because it complicates surveillance for LTBI (Question 10).

It is well-established that LTBI and active TB are more common among foreign-born individuals, and that foreign-born students are more likely to have received BCG vaccine than students born in the U.S. [17]. To evaluate these factors, "Were you born outside the U.S.A. or Canada (are you foreign born)?" was asked as a general question. Among the 1469 students who responded to this question, almost one-quarter (22.9%) reported they were born outside the U.S.A. or Canada. Of the foreign-born, the majority (72%) were in medicine and pharmacy programs. As expected, a significantly higher proportion of foreign-born students had been diagnosed with LTBI (14.9% vs. 3.9%), had been treated for LTBI (6.5% vs. 1.7%), and had received BCG vaccine (33.6% vs. 1.3%).

Table 3 shows general beliefs about TB education. The majority (90.5%) disagreed with the statement, "There is only minimal need for more education on TB because it is not likely that I will need it in my chosen career," and with the statement, "The career path I have chosen will not require me to know much about TB" (85.8%). The majority (85.7%) agreed, "TB education is very important in my academic program." The majority, although to a lesser extent (70.0%), agreed "The current emphasis on TB in my academic program is adequate". There was more ambivalence (agreement = 61.3% vs. disagreement = 38.7%) about the question, "In my future plans as a health professional, I am confident that the level of TB knowledge I have attained is adequate to prepare me for my career needs."

**Table 3:** General beliefs about tuberculosis education from NTCC Survey, 2005 (N = 1480). Responses were recorded on a 4-point Likert-type scale (Strongly Disagree = 1, Disagree = 2, Agree = 3, Strongly Agree = 4). For this table, responses have been combined into two categories: Disagree (SD + D) and Agree (A + SA).

**Statement**	**4 point mean**	**Disagree N (%)**	**Agree N (%)**
*1. There is only minimal need for more education on TB because it is not likely that I will need it in my chosen career*.	1.66	1388 (90.5)	140 (9.5)
*2. TB education is very important to my academic program*.	3.16	211 (14.3)	1267 (85.7)
*3. The current emphasis on TB in my academic program is adequate*.	2.74	444 (30.0)	1038 (70.0)
*4. The career path I have chosen will not require me to know much about TB*.	1.76	1267 (85.8)	209 (14.2)
*5. In my future plans as a health professional, I am confident that the level of TB knowledge I have attained is adequate to prepare me for my career needs*.	2.62	573 (38.7)	906 (61.3)

Table 4 shows the number of patients with LTBI and active TB cared for by students during their academic program to date. A substantial proportion had cared for no LTBI or TB patients during their program, varying from a low of 26.8% among medical students to a high of 75.5% among pharmacy students. Most students who had cared for at least one patient had cared for fewer than 4 patients. About 20% of NP/APN and RT students had cared for more than 6 patients.

**Table 4:** Patient care experiences reported by respondents to NTCC Survey, 2005. Number of patients with latent tuberculosis infection (LTBI) or active tuberculosis (TB) cared for during their academic program to date as reported by students in six clinical disciplines (clinical laboratory sciences and public health students did not answer this question).

**Response**	**Medicine** **MD/DO** ** N (%)**	**Physician Assistants** ** PA** ** N (%)**	**Under-graduate Nursing** ** BSN** ** N (%) **	**Advanced Practice Nursing ** **NP/APN** ** N (%)**	**Pharmacy** **PharmD** ** N (%)**	**Respiratory Therapy ** **RT** ** N (%)**	**Total ** **N (%)**
**None**	130(26.8)	47 (47.0)	139(55.4)	38(41.3)	275(75.5)	16(38.1)	645 (48.5)
**1–3 patients**	246(50.7)	36 (36.0)	83 (33.1)	28(30.4)	63 (17.3)	15(35.7)	471 (35.3)
**4–6 patients**	65 (13.4)	5 (05.0)	11 (4.4)	6 (6.5)	12 (3.3)	3 (7.1)	102 (7.7)
**> 6 patients**	42 (8.7)	12 (12.0)	18 (7.2)	19(20.7)	14 (3.8)	8 (19.0)	113 (8.5)
**Total (% of respondents)**	483(36.3)	100 (7.5)	251(18.9)	91 (6.8)	364(27.3)	42 (3.2)	1331*

* Three students did not answer this question

## Discussion

In a recent editorial, Jensen [18] commented on a study of medical students in Brazil [19]. Both authors strongly recommended providing better education about TB to medical and other health professions students. Teixeira et al. [19] found that 10.3% of medical students did not understand that *M. tuberculosis *is transmitted by coughing (24.3% of students in pre-clinical years and 2.4% in late clinical years). This is similar to our overall findings wherein almost 10% did not know TB organisms were most commonly transmitted from person-to-person via aerosols. Among our medical students, however, most of whom were near completion of their training, 95% knew the correct answer which is more consistent with Teixeira et al.'s findings for medical students in their late clinical years.

We were pleased that most students believed TB education was important to their academic program (Table 3, Question 2) and that their career path would require knowledge of TB (Table 3, Question 4). However, the fact that 30% of students disagreed the current emphasis on TB in their academic program was adequate (Table 3, Question 3) is consistent with finding that four of the five basic TB questions were answered incorrectly about one-third of the time (Table 2, Questions 7–10), and almost 40% of the respondents were not confident their level of TB knowledge was adequate for their career needs (Table 3, Question 5).

Our findings are also similar to those of several other investigators who have conducted surveys among health professions students, primarily outside of the U.S.A. For example, Kilicaslan et al. [20] evaluated undergraduate training on TB at Istanbul Medical School and found among fourth-year medical students (n = 828) many incorrect answers. They also reported examination questions did not adequately reflect WHO learning objectives for medical schools, published in 1998 [21]. Kurane and Kudoh [22] administered two sets of questionnaires to physicians in 80 medical school hospitals in Japan in 2002. They concluded additional education was needed for physicians and medical students. Bai et al. [23] administered a survey to final year medical students in Hunan province of China in 2000 and came to similar conclusions. In contrast, Emili et al. [24] assessed final year medical students from Canada, India, and Uganda. They concluded although there were significant differences in undergraduate exposure to TB, total knowledge, and practice competency among these students, the TB knowledge base and practice competency was adequate.

In our study, students received TB education primarily from lectures and case discussions or case studies. Lectures are a passive learning modality that does not appeal to many of today's learners who prefer more active strategies [25,26]. Although case discussions and case studies are types of active learning used by almost half of the students (46%), other more active strategies such as standardized patients, games, and computer simulations were used much less frequently. An NTCC objective is to develop educational materials using active-learning strategies. Several products are now available on the NTCC website [14] by NTCC and other faculty. In addition, NTCC faculty are currently integrating NTCC-developed materials into their curricula for the 2007 and 2008 academic years and will survey students again in Spring 2008 to determine whether these materials have had any impact on their knowledge, attitudes, and confidence in caring for patients with LTBI or TB.

### Limitations of the study

There are several limitations to our study. First, the surveys were administered to students in only 28 programs among the hundreds of programs in the United States of America. Most NTCC faculty are affiliated with academic institutions in high TB incidence states, and a few are from low TB incidence states, but the annual incidence of TB in the United States is overall quite low (fewer than 15,000 new cases in 2005 [5]). Accordingly, many students had little or no experience caring for LTBI or TB patients. Second, the response proportion in the seven clinical disciplines was quite good (almost 65%) for a voluntary survey; however, we still sampled fewer than 1500 of the many thousands of health professions students in the U.S.A., making it inappropriate to generalize these results to all health professions students. Third, survey questions were developed by the NTCC from a variety of sources because a standardized survey instrument was not available. Throughout the survey development process, questions were reviewed and modified several times, and consensus was gained by each discipline group for each final version; formal instrument development was not done. Similar to test analyses conducted by UCSD EdCom for multiple-choice tests administered in academic courses, a test analysis was conducted for the five knowledge questions (Table 2: questions 6–10). The test analysis showed that these questions had adequate to good discrimination, efficiency, and correlation indices (B. Stanonik, personal communication); however, we would have greater confidence about their validity and reliability had we conducted a formal instrument development procedure.

## Conclusion

The NIH-NHLBI funded the NTCC to develop products to improve knowledge of TB in health professions students in the U.S.A. based on the assumption that there was a need. This study supports that need by demonstrating considerable room for improvement in knowledge, attitudes, and confidence about TB among health professions students surveyed in 28 programs in 20 schools in the U.S.A. Information about learning styles and preferences of current students supports the belief that active-learning strategies may improve student performance. This belief will be assessed with future surveys of students in NTCC schools who have been exposed to NTCC-developed educational products that use active-learning methodologies.

## Competing interests

The author(s) declare that they have no competing interests.

## Authors' contributions

All authors were responsible for the conception and design of the study, and for development and refinement of the survey instrument. MJ analyzed the data and drafted the manuscript. All authors participated in review and revision of the manuscript. All authors read and approved the final manuscript. All authors confirm that the content has not been published elsewhere and does not overlap or duplicate their published work.

## Appendix

NTCC Participating Schools

**Administration: **University of California, San Diego

Curriculum Centers for each Region are in ***bold italics***

Western Region:

• ***University of Southern California, Los Angeles***

• University of California, Berkeley

• Stanford University, Palo Alto, CA

• Western University, Pomona, CA

• University of Washington, Seattle

• WA State Department of Health, Seattle

• University of Colorado, Denver

Northeast Region:

• ***Columbia University, New York, NY***

• Long Island University, Brooklyn, NY

• Northeastern University, Boston, MA

• NY College of Osteopathic Medicine, Old Westbury

Southeast Region:

• ***University of Arkansas, Little Rock***

• Georgia State University, Atlanta

• Johns Hopkins University, Baltimore, MD

• University of Maryland, Baltimore

North Central Region:

• ***Wayne State University, Detroit, MI***

• University of Illinois at Chicago

• University of Michigan, Ann Arbor

• Midwestern University, Downers Grove, IL

• Rush University, Chicago, IL

• University of Nebraska, Omaha

South Central Region:

• ***University of Texas Health Science Center, Houston***

• University of Texas, San Antonio

• Tulane University, New Orleans, LA

## Pre-publication history

The pre-publication history for this paper can be accessed here:


